# Supplementary material for: Pesticide-induced resurgence in brown planthoppers is mediated by action on a suite of genes that promote juvenile hormone biosynthesis and female fecundity
Source: eLife. 2025 Sep 30;12:RP91774. doi: 10.7554/eLife.91774 (PMC12483516; doi:10.7554/eLife.91774)
Supplement: Supplementary file 1. [file elife-91774-supp1.docx]

**Supplementary File 1.** Determination of the toxicity of emamectin benzoate on BPH in systemic and topical application bioassays.

| Treatment method | Developmental stages | Slope ± SE | LC_15_ (95%F.L.) (mg/L) or LD_15_ (95%F.L.) (pg/insect) | LC_50_ (95%F.L.) (mg/L) or LD_50_ (95%F.L.) (pg/insect) | *χ*^2^(df) | *P value* |
| --- | --- | --- | --- | --- | --- | --- |
| Systemic route | 4^th^ nymph | 2.56 ± 0.487 | 0.87  (0.39-1.32) | 2.21 (1.53-2.82) | 2.68 (4) | 0.61 |
|  | Adult Male | 2.42 ± 0.35 | 0.80  (0.40-1.20) | 2.13 (1.48-2.78) | 2.12 (4) | 0.72 |
|  | Adult Female | 2.47 ± 0.41 | 2.33  (1.09-3.56) | 6.12 (4.18-8.00) | 3.56 (4) | 0.50 |
| Contact route | 4^th^ nymph | 2.52 ± 0.35 | 15.54 (9.46-21.42) | 39.90 (30.66-49.56) | 2.60 (4) | 0.63 |
|  | Adult Male | 2.77 ± 0.57 | 6.72  (2.52-10.92) | 15.96  (9.66-21.84) | 1.23 (3) | 0.75 |
|  | Adult Female | 3.31 ± 0.76 | 18.48 (6.72-28.14) | 37.80  (23.10-50.40) | 0.79 (3) | 0.85 |
