# Supplementary material for: Pesticide-induced resurgence in brown planthoppers is mediated by action on a suite of genes that promote juvenile hormone biosynthesis and female fecundity
Source: eLife. 2025 Sep 30;12:RP91774. doi: 10.7554/eLife.91774 (PMC12483516; doi:10.7554/eLife.91774)
Supplement: Supplementary file 2. [file elife-91774-supp2.docx]

**Supplementary File 2** Sequences of oligonucleotide primers used in this study.

| Primers | Primer sequences (5’ - 3’) |
| --- | --- |
| **For cDNA cloning** | |
| **Allatotropin：** | |
| NlAT-F | TACGCGGCCAAACACACTTA |
| NlAT-R | AGGGAAAGAGGGCGAAATTCA |
| **Allatostatins：** | |
| NlAstA-F | TCGGCCGTCACAAGTCAAG |
| NlAstA-R | CCGAACCCGTACTTCATGCT |
| NlAstB-F | ACCGGGCTCACAGGAATTTT |
| NlAstB-R | TGTAGGCGCAGATCTTGAGG |
| NlAstCC-F | AACACAGCTCTACGAGGCAC |
| NlAstCC-R | CCAAGCAGGTGACTGCCATA |
| NlAstCCC-F | TTTGTGTGTGCTTGCAGGTG |
| NlAstCCC-R | GGATAGAAACGGTAGATTTGGTAGA |
| **Allatostatins receptor：** | |
| NlA16-F | CCTCATTGTGGAACCACCGA |
| NlA16-R | CGCAGCTGTAAGGTGGAAGA |
| NlA2-F | GAACGTAATGGGAGTCGGCA |
| NlA2-R | GTTTTTGTGAGCGCCGACTT |
| NlA10-F | ATGCAAAACACGGCCAGCCT |
| NlA10-R | TTAATCGTCTCTGCTCAACTCCAAAGGAAGGT |
| NlA1-F | CGACCAGACCACTCTACTGC |
| NlA1-R | ACGTGGACCTCACTATACCAAAAA |
| **For Quantitative RT-PCR** | |
| Q-Nl18S-F | CGCTACTACCGATTGAA |
| Q-Nl18S-R | GGAAACCTTGTTACGACTT |
| Q-vitellogenin-F | GTGGCTCGTTCAAGGTTATGG |
| Q-vitellogenin-R | GCAATCTCTGGGTGCTGTTG |
| Q-Vitellogenin receptor-F | AGGCAGCCACACAGATAACCGC |
| Q-Vitellogenin receptor-R | AGCCGCTCGCTCCAGAACATT |
| Q-JHE-F | GAGCCTCACATCCACAGC |
| Q-JHE-R | AATGGGAGCCCTACGC |
| Q-NlMet-F | GGTGGTAAACGGATTGGAAA |
| Q-NlMet-R | CATCGTCAGCCAACTCGATA |
| Q-JHAMT-F | GAACCTGCAGGCCAAACACA |
| Q-JHAMT-R | ACCACTCGGTTGGGCTGAAT |
| Q-NlKr-h1-F | TGATGAGGCACACGATGACT |
| Q-NlKr-h1-R | ATGGAAGGCCACATCAAGAG |
| Q-NlAT-F | CACGATACGTGGCTTCAAGA |
| Q-NlAT-R | ACGATCACTTTCGCCAATTC |
| Q-NlAstA-F | AGGACTTACTGGGCGAGGAT |
| Q-NlAstA-R | GGTGTCTCGTTTCCTGGTGT |
| Q-NlAstB-F | AGCGAGCTAGACGAGGACAA |
| Q-NlAstB-R | TCGTCTCTGCTCAACTCCAA |
| Q-NlAstCC-F | CTGCTCCCAGTGAAAAGGAG |
| Q-NlAstCC-R | GCTTCCAGTAACTGCGCTTC |
| Q-NlAstCCC-F | TGTGCTTGCAGGTGGTAGTC |
| Q-NlAstCCC-F | AGAAGCATGTGACTGCGTTG |
| Q-NlA2-F | TCCTGGTGCTGAAGAGTGTG |
| Q-NlA2-R | CTTTTCGGGCCCATTAATTT |
| Q-NLA1-F | ATATCGGCACCGAAGATGAG |
| Q-NLA1-R | GTCTGACCCGACAGGTTCTC |
| Q-NLA10-F | ACTGGGTGTCGACCAATCTC |
| Q-NLA10-R | TCGGTAGCGAGGAAGACAGT |
| Q-NLA16-F | TACCGTTCTGTGGGATGTCA |
| Q-NLA16-R | CCGGGATATCAAAGACGAGA |
| Q-NlGluCl-F | CACTGACTGAGGCCAACAGA |
| Q-NlGluCl-R | GCTGGCCATTCTTAGTGAGC |
|  |  |
| **For double-stranded RNA synthesis** | |
| T7-EGFP-F | TAATACGACTCACTATAGGGCGTAAACGGCCACAAGTTCA |
| T7-EGFP-R | TAATACGACTCACTATAGGGGACTGGGTGCTCAGGTAGTG |
| T7-Kr-h1-F | TAATACGACTCACTATAGGGCGCCAGTGAAAGTGAGACCT |
| T7-Kr-h1-R | TAATACGACTCACTATAGGGGAGACCGCAAGTGGTTCTGA |
| T7-Met-F | TAATACGACTCACTATAGGGCCACCAACCAGCAGATGAACCTGA |
| T7-Met-R | TAATACGACTCACTATAGGGCCACGCAAAGCCTCGTACTCTTGG |
| T7-JHAMT-F | TAATACGACTCACTATAGGGCTCCAGGCCATTGTCCCTCA |
| T7-JHAMT-R | TAATACGACTCACTATAGGGTTGGCCTGCAGGTTCTTTGG |
| T7-AstA-R-F | TAATACGACTCACTATAGGGTACTGCCGTTCTGGCCTTTT |
| T7-AstA-R-R | TAATACGACTCACTATAGGGGTGATCTGGAAGAGCGGCTT |
| T7-Glucl-F | TAATACGACTCACTATAGGGACACATCACCTGCTCACCTG |
| T7-Glucl-R | TAATACGACTCACTATAGGGGTGTGTTTGCCTGCTGTCTG |
|  |  |
