# Supplementary material for: Pesticide-induced resurgence in brown planthoppers is mediated by action on a suite of genes that promote juvenile hormone biosynthesis and female fecundity
Source: eLife. 2025 Sep 30;12:RP91774. doi: 10.7554/eLife.91774 (PMC12483516; doi:10.7554/eLife.91774)
Supplement: Supplementary file 3. [file elife-91774-supp3.docx]

**Supplementary File 3 Amino acid sequences of neuropeptide G Protein-Coupled Receptors (GPCRs) and related receptors from multiple arthropod species**

> neuropeptide GPCR A1 Nilaparvata lugens BAO01050.1

MELPFEEGFTLGMSPLNGSLDNETLLGNTTFCKTIDQTTLLLFTQVLYAIVCIVGLFGNSLVIYVVLRFSKMQTVTNMYIVNLAVADECFLIGIPFLIATMSLQLWPFGKIMCKFYMTTTSINQFTSSIFLTIMSADRYVAVCHPISAPKMRTPFISKVVSATAWTASALLMIPIFMYASLMNDGQVNMSCNILWPESENLSGQTAFTLYSFVLGFALPLLLIFCFYVMVIRKLKTVGPKNKSKEKKKSHRKVTKLVLTVITVYVCCWLPYWITQMSLIFTEPNKCQSRFVVTVFLLTGILCYSNSAMNPILYAFLSDNFKKSFLKACTCAAGKDVSRIKLQINTIINLLKYYIFWYSEVHVIQ

>neuropeptide GPCR A1 Bombyx mori NP_001127736.1

MEIEEIELYRQMNYSYDFNGTFNGTMGTCPIVNLPYVSIVTQVLYALVCIVGLLGNTLVIYVVLRYSKMQTVTNMYIVNLAIADECFLIGIPFLITTMSLNKWPFGDYMCKTYMISTGINQFTSSIFLCIMSADRYIAVC

HPIAAPRLRTPCVSRIVSAAAWTASAAIMTPIFMYAKLVRIGNKLSCNIVWPEQDFSQGQITFTLYSFALGFAAPLTLIFIFYCLVIRKLKTVGPKNKSKEKKRSHRKVTKLVLTVIAVYVLCWLPYWAFQMALIYSPPS

QCVNHITITVFLVAACFSYSNSAMNPILYAFLSDNFKKSFLKACTCAAGKDVNATLHVENSVIPRRRARAQARAAEARGGFAAAVGGSRSEASTAMTSRSMAASEVLPLEARPPTLTPLIAHNGLSHSRL

>neuropeptide GPCR Tribolium castaneum 52 NP_001280528.1

MFRTRDSTSAMIIFSDSNDDMGIVFTLEGLTQAGIIFIMAIAIIVTNILIIATFLNFRGPSEVINCYLLS

LAVADLLCGLLVVPLSVYPAVVREWVYGDIVCRLVGYLEVTLWAVTVYTFMWISVDRYLAVRKPLRYETL

QTKTRCQCWMAFTWISAAMLCCPPLLGFNQPVFDKDAYICMLDWGSMAAYSVTLSILVLGPSLITIVYTY

TYIFNMLRKLRSGYAFHDKEYATALSENLSNPSHLMSFSLVVAFWVSWTPYIGVKLYEYFTGVKLQVQFL

HFGIVWLGFLNSFWKSMILITMSPQFRLALRIFCMTICCRYKGRMQAELIGMEADD

>allatostatin C R1 Drosophila melanogaster NP_649040.2

MFTWLMMDVLQFVKGEMTADSEANATNWYNTNESLYTTELNHRWISGSSTIQPEESLYGTDLPTYQHCIA

TRNSFADLFTVVLYGFVCIIGLFGNTLVIYVVLRFSKMQTVTNIYILNLAVADECFLIGIPFLLYTMRIC

SWRFGEFMCKAYMVSTSITSFTSSIFLLIMSADRYIAVCHPISSPRYRTLHIAKVVSAIAWSTSAVLMLP

VILYASTVEQEDGINYSCNIMWPDAYKKHSGTTFILYTFFLGFATPLCFILSFYYLVIRKLRSVGPKPGT

KSKEKRRAHRKVTRLVLTVISVYILCWLPHWISQVALIHSNPAQRDLSRLEILIFLLLGALVYSNSAVNP

ILYAFLSENFRKSFFKAFTCMNKQDINAQLQLEPSVFTKQGSKKRGGSKRLLTSNPQIPPLLPLNAGNNN

SSTTTSSTTTAEKTGTTGTQKSCNSNGKVTAPPENLIICLSEQQEAFCTTARRGSGAVQQTDL

>allatostatin C R2 Drosophila melanogaster NP_001027135.2

MEGGWWRGGGGGGRLGGKAIMEGHSTPNGAAASHRNNSTRTNIATNGCAHSGILLFVLTAMTLTSLITPT

EQLAVAPNGTTLHQLESVESESYPSINGTQNETMVTSVRPHLDHRNRPTQQNGSHYLEYDDDGPDCSYSY

NFILKLITMILYALVCIIGLFGNTLVIYVVMRFSKMQTVTNIYILNLAIADECFLIGIPFLLYTMQVGNW

PFGNYMCKAYMVSTSITSFTSSIFLLIMSADRYIAVCHPISSPRYRTPFVSKLVSAFAWMTSVLLMLPVI

LFASTVQSSNGNVSCNIEWPDTQNSHTDSTFILYSLVLGFATPLTFILVFYCLVIRKLHTVGPKHKSKEK

KRSHRKVTKLVLTVISAYIFCWLPHWISQVALISSAPQRCASRLELAVFLACGCLSYSNSAMNPILYAFL

SDNFKKSFMKACTCAARKDVNAQLQLENSFFPKFGKGRQSERLLGGNGKGGAQRGALTKKKCLATRNNNA

PMATTTTTTTTTTGTDAVTCLQPPVHQVPAEIQVGNPATVLVVNAETNNCKPPVLHTDL

>neuropeptide allatostatin CR Tetranychus urticae XP_015794199.1

MDTSKLLNDSIYIPPDGFNNIGLIPSSSNISAFIEFQGFGKNDTSYADRLDLMKKLEEIKVNRVIDQDAL

IWLMLSYILLIAVGSIGNGLVCIAVIRKPSMRTPRNLFIINLAISDLTLCLITMPFSFIEIAAKFWSLGL

FTCKLIAGLEATSIFVSTMSITAIALDRYFVIIKPTPETPKITGILYGLLSIWIIALILSIPLFWSRTTY

RIEVPEILSSADHSKETLEYCYEEWPVNRGRAIYSVFTIVLQYFVPTILVTLIYMKIYKRLKNRMSQKRT

AIKLDERIKAEERRTKRTNYLLISVSLIFGISWLPLNILNIISDVYYPFQDTSTFRIVFACCHMVGMSSA

CFNPLLYGWLNDNFQKEFKEIFALITGKLSSCCSVKGSIISGRTSISLEGRIETTVVYGESHDKESNHLQ

HMHHKSSDTDHNTGNCNTNSDTSNGNGSAAKKSLVVKQV

> AstA-R1 transcriptome GPCR A2 [Nilaparvata lugens] BAO01051.1

MAVPPNVMGVGIPVFMYNNCLNLTNLSEVAFCQNASSISTPEDNGDPDSFTVMEKIVSIAVPILFGIIVLLGLFGNLLVVIVVAANQQMRSTTNLLIINLAVADLLFIVFCVPFTATDYILPFWPFGDFWCKTVQYLICVTAYASVYTLVLMSLDRFLAVVHPIASMSVRTERNAITAIVVVWVVIVVGCVPVFLSHGVASYVYSSNVQSACVYLQYDPVNRPDGHNKPLFQITFFTTSYVVPLALICGLYLCMIMRLWRGVAPGGHCSAESRRGKKRVTRMVVVVVAIFAICWCPIQVILVLKSVDRYEITNTSVMVQIVSHVLAYMNSCVNPILYAFLSENFRKAFRKVIYCGPDRAHMTGQINGPEKSALTKTTRTNDIL

> allatostatin-A receptor [Bombyx mori] NP_001037035.1

MESTEDEFYTICLNLTAEDPSFGNCNYTTDFENGELLEKVVSRVVPIFFGFIGIVGLVGNALVVLVVAANPGMRSTTNLLIINLAVADLLFVIFCVPFTATDYVMPRWPFGDWWCKVVQYFIVVTAHASVYTLVLMSLDRFMAVVHPIASMSIRTEKNALLAIACIWVVILTTAIPVGICHGEREYSYFNRNHSSCVFLEERGYSKLGFQMSFFLSSYVIPLALISVLYMCMLTRLWKSAPGGRVSAESRRGRKKVTRMVVVVVVVFAVCWCPIQIILLV

KALNKYHITYFTVTAQIVSHVLAYMNSCVNPVLYAFLSENFRVAFRKVMYCPPPYNDGFSGRPQATKTTRTGNGNSCHDIV

> allatostatin-A receptor1 [Drosophila melanogaster] NP_524700.1

MAGHQSLALLLATLISSWPKASWGATGNGSIISVSNSSGNNYAFTSEHTDHSDHNANDSMEYDAESVALERIVSTIVPVFFGIIGFAGLLGNGLVILVVVANQQMRSTTNLLIINLAVSDILFVIFCVPFTATDYVLPEW

PFGNVWCKFVQYMIVVTCHCSVYTLVLMSFDRFLAVVHPVTSMSLRTERNATLAIMCAWITIVTTAIPVALSHSVRIYQYHGNAGTACVFSTEEEIWSLVGFQVSFFLSSYVAPLTLICFLYMGMLARLWKSAPGCKPSA

ESRKGKRRVTRMVVVVVLAFAICWLPIHVILVLKALNLYGGSHLSVIIQIISHVVAYTNSCINPILYAFLSDNFRKAFRKVVWCGSPPPLMTNQQVTKTTRTATGNGTSNIEML

> allatostatin-A receptor2 [Drosophila melanogaster] NP_524544.1

MENTTMLANISLNATRNEENITSFFTDEEWLAINGTLPWIVGFFFGVIAITGFFGNLLVILVVVFNNNMRSTTNLMIVNLAAADLMFVILCIPFTATDYMVYYWPYGRFWCRSVQYLIVVTAFASIYTLVLMSIDRFLAV

VHPIRSRMMRTENITLIAIVTLWIVVLVVSVPVAFTHDVVVDYDAKKNITYGMCTFTTNDFLGPRTYQVTFFISSYLLPLMIISGLYMRMIMRLWRQGTGVRMSKESQRGRKRVTRLVVVVVIAFASLWLPVQLILLLKS

LDVIETNTLTKLVIQVTAQTLAYSSSCINPLLYAFLSENFRKAFYKAVNCSSRYQNYTSDLPPPRKTSCARTSTTGL

> allatostatin-A receptor [Tetranychus urticae] XP_015789363.1

MDPPGINNIFSQLRTLYSPSPSSSHLPSSPLSDSSEEPLYLDHLSVDPSLNSTGTSFCGSLISSLDPNVS

QIYADECSFANVEEVVRILVPLVFSIIVVIGLLGNSLVVIVVLWDDQMRSTTNVLIFNLAVADLLFIIFCVPFTATDYALKYWPFGDIWCRIVQYLIYVSAFASIYTLVLMSLDRFLAVVHPIASLSIRTEVNAYRAITI

LWIAIIFLCLPILIVHQVHVDLDSETSHLCHINPAENKFFAPSVFLSSYAIPLTLAFVLYVYMLKRLWHNDIRTGRSVRNKRKVTRMVVVVVVIFAICWGPIHIVLTLRSQGMFEPDSKFKLVTLIVSQILAYINSCINP

ILYAFLSENFRKAFRKVISCRPSGSRRRHDRPGSAKEETNRMDELTTATCQTTKRTELTNGCV

> MIPR/AstBR BAO01060.1 neuropeptide GPCR A10 [Nilaparvata lugens]

MYPTMSNYILIEYSNSSDGGAASLNFTTLLPPNITLDSNLTADQLIPDYLNVTRELPIQYAQPMYGYVMP

LLLLITIVANTLIVVVLSKRHMRTPTNAVLMAMALSDMFTLLFPAPWLFYMYTFGNHYKPLSPVGACYAWNVMNEVIPALFHTASIWLTLALAVQRYIYVCHAPVARTWCTMPRVLKCVAWISVLATLHQSTRFVDRVYEPLTIKWRDQEHVTVCQVRIAYWVEHWVSTNLYFTLYFCFRVIFVHAIPCVCLVALNVLLFRALRVAQLKRDKLFKENRKSECKRLRDSNCTTLMLIVVVTVFLATEIPLAVVTVLHIISSSITEILDYSVANVLVLFTNFFIIVSYPINFAIYCGMSRQFRETFKELFMRGAVTVARRNGGAGGSSRYSLVNGPRTTTNETVL

> sex peptide receptor [Tribolium castaneum] NP_001106940.1

MGEMASNSTLIFPNQTYANETVPNVTSVEKVQYINITMEMPIAYAVPLYGYVMPFLLIITIIANTLIVVV

LSKRQMRTPTNVVLMAMALCDMFTVLIPAPWLIYMYSFGNHYKPLWPISLCYAWFVMHEVIPNMFHTASIWLTLALAVQRYIFVCHAPLARKLCTMSNVYKCLIYILVIAALHQGFRFFDSEYSTVDVLWNNHTTHVCKREHAHWVKEYVTEDFYFVTYFMFRVLFVHLIPCVALVILNILLFRALKQAQQRREQLLSKKNQKNECKKLRDSNCTTLMLIVVVTVFLIVEIPLVVVTLLHIISSTFIEFLDYYVANTLILFTNFFIILSYPINFAIYCGMSRQFRETFKELFIRGAVTTRNGSSRYSLVNGPRTCTNETVL

>sex peptide receptor [Bombyx mori] NP_001108346.1

MAVTIDNSTNDFEFQKPFNYSINENITYFDYTNFTSDDFCASNNSHVYLNVTCEFAISYAEPMYGYIAPF

LLATTTVANTLIVVVLSRRHMRTPTNAVLMAMALCDMFTMLFPAPWLFYMYTFGNHYKPLSPVRACQAWNYMNEVIPAMFHTASIWLTLALAVQRYIYVCHAPVARTWCTMPRVMKCLIYIGIAAFLHQLPRFFDRCYTPHKTVWRGRVEEVCRIEMASWVKALSVDAYFISYFGFRVLFVHLIPCTSLVVLNVLLFRAMRTAQINRQKLFKENRKSECKRLRDSNCTTLMLIVVVTVFLLVEIPVAVVTILHIISSTIVEILDYHIANILVLVTNFFIIVSYPINFAIYCGMSRQFRETFKELFIRGTVTSRKNGGSSRYSLVNGPRTCTNETVL

>SPR [Drosophila melanogaster] NP_001368972.1

MDNYTDVLYQYRLAPSASPEMEMELADPRQMVRGFHLPTNESQLEIPDYGNESLDYPNYQQMVGGPCRME

DNNISYWNLTCDSPLEYAMPLYGYCMPFLLIITIISNSLIVLVLSKKSMATPTNFVLMGMAICDMLTVIF

PAPGLWYMYTFGNHYKPLHPVSMCLAYSIFNEIMPAMCHTISVWLTLALAVQRYIYVCHAPMARTWCTMP

RVRRCTAYIALLAFLHQLPRFFDRTYMPLVIEWNGSPTEVCHLETSMWVHDYIGVDLYYTSYYLFRVLFV

HLLPCIILVTLNILLFAAMRQAQERRKLLFRENRKKECKKLRETNCTTLMLIVVVSVFLLAEIPIAVVTA

MHIVSSLIIEFLDYGLANICIMLTNFFLVFSYPINFGIYCGMSRQFRETFKEIFLGRLMAKKDSSTKYSI

VNGARTCTNTNETVLXXLVMLVPRRGSSDHRRSSTSTTTTTTTKTIGGSMIIGGEASAQHQHLVTHHLQT

HSQPSQQRRVSTMDIITEERIL

>SPR [Tetranychus urticae] XP_015790938.1

MRVEPWVEFYFYGVLLTSMCLFGISANTVTLFILNAFNEMRRQPINVYLTVLAIYDNGVLINALLMLGIP

ALVTNSKHNPSDYLPSVFPSLDSSSPSSISPVNSLHPSSSSSPLSSSFSTSSSPLPVSPHLSSSLPNQLS

SVNLPSSESSELEKSQSTDGSTDSMRLTDNTFDYTSTDYLNRLQSLDPYSVNLTLMNKSHSWTPTELINL

NPSSIDPPLLFDLSLAHGYSNPEDLPPLVIPPISHSTPTNHHHHQHSLPPSPGYSTELPDDPLNYYVTFV

YPLALISQTGSIWTTCLITAERYFAVCHPFRIRTFSNRNRAIWAVVFLSLGAFLYNIPRFIEIEVITSPE

GIRSVRQTALRQNRLYYWLYYICLNLALLYIIPLSLLTALNTEIYKAVRRASRNRATLTNQEETELNIAS

MLVLLVSIFIACNAPAFVVNCMEFFNAPGYEMATIFSNLLVCLNSSINFVIYCIFGKKFRKKLSQVFHCS

KLAHQDRLANSYQARVNVTTTALNANLIVKNTVNDEQNNSNNVNLVNNISIATTNSMHAINETYV

>A16 transcriptome [Nilaparvata lugens] BAO01066.1

MMFVVVADFDSDLRFDFNNPFNDSYIYDAKGIDTWENIHYESMTKRTLIVEPPRLLESSSQQTGVAVQKPGSSDAKKMDNSAGVKDLVLLDTKDSEPTTNATNGTTAVGNSTLDDNCSNDYCISDQDYYDMIVQHILPSKSECLLIALHGVVFCVGLVGNALVCLAVYRNRSMRTVTNYFIVNLAVADFMVILFCLPPTVLWDVTQTWFMGTLVCKIVLYFQTVSVAVSVLTLTFISVDRWYAICFPLKFKSTTGRAKTAILIIWLLALVFDIPELLVLEAQKFGKFKEIDMDLIFFTQCQATWSEGTEGMYQMARILLLYTFPLMFMSIAYCQIVIVLWRSDNIPGHSETVLVHSGNGHGGKGRVANNSTECQLRSRRKAAKMLVAVVVMFAVCYFPVHLISILRMTMDIKQTEVTGGLAIVSHWLCYANSAVNPVIYNFMSGKFRKEFQRAFGNVFKRKPSQRTTRRGESTLGCRYTTMSAGTHTTPKTEIVQLNTTVVTINEIPEQSARATTPATAPATATATAPTTAPATAPRTAAVALNRAHRALCRQDISPRQGRIQRIGLEEKGCIFEMRNITEEQQDADLEEIVIDRG

>neuropeptide receptor A5 [Bombyx mori] NP_001127740.1

MALRKESLAIITMLIICNYVLSSNFDSIPESIRVRKSVDNTTSRSSLKNLNETMKQSNNETEFGRLLDATEMTTEYDNFTEEPCVGDRAFCNLTREEYMEMLNDYVFPQPYEWVLIATHAIVFVIGLIGNALVCIAVYRNHSMRTVTNYFIVNLAVADFMVILICLPPTVLWDVTETWFFGTAMCRIVLYFQSVSVTVSVLTLTFISVDRWYAICFPLKFKSTTGRAKTAILIIWLLSLLFNIPEFVVLQVQTKMQLRFNVQYFMQCASTWSDESDLTWHIIKALFLYTFPLLLMTIAYCQIVRVLWRSDNIPGHTESHKLCSTQTGQSNWLAASRRTTPSIHTNASTEGQLRSRRKAAKMLVAVVAMFAVCYFPVHLLSVLRVAFDVQQTDVMTCIALISHVMCYANSAVNPLIYNFMSGKFRREFHRSYFKCFCCCHTTPAPEQNGASFEPIGSSRARTIRTTVRRHDSCVSYRLAHLSPSNHNIHRDYIQNTNTSFIEPMNGNRRSKIRDESISDTATRFTVTTDIPCKD

>neuropeptide receptor A16 [Bombyx mori] NP_001127714.1

MTTVEDDLNVPKKMKANKIISEHDDRFKTDTNSSEFEEAENETCVGDPQYCNMTKEEYVKMIQEYIYPNPYEWILIATHTFVFITGLFGNALVCVAVYRNHSMRTVTNYFIVNLAVADFMVILFCLPATVLWDVTETWFL

GEGLCKVLPYFQSVSVTVSVLTLTFISVDRWYAICFPLKFKSTTGRAKTAILIIWLVSLCFNIPELVVLKLVRFVPLRFELPYLLQCYGTWSPSSELVWHILKVLLIYTLPLVLMAVAYHQIARVLWSSNGIPGQADTKK

LATAELTQLRSRRKAAKMLVSVVIMFAVCYFPVHLLSVMRYTIDMGQTEFITIWALVSHVMCYANSAINPLIYNLMSDKFRREFRRAFCCSTSPGQQDFTSMSRVTTKKDSSIMASFKPGHTSTTFVHNNKNGHMT

>neuropeptide receptor TC37 [Tribolium castaneum] XP_973738.2

MLFFLLATILLSHAQAHDGLTSPHERANNSLFVSKPRPRNDTFIDDQFDYLVRDKRDWDEDNASYINGSGNVTFSEQEFIDSLWELIAPKSWTWILVILHSLVFIIGIIGNILVCVAVYRNHTMRTVTNYFIVNLAVADF

LVILFCLPPSVVWDVTVTWFFGVTMCKIVLYFQSVSVTVSVLTLTFISIDRWYAICFPLKFKSTTGRAKTAIGIIWIVALACDIPEMIYVTTIPTVDEVDTVLLTQCAPTWSTETDTIFFILKMVLFYLIPLLFMSIAYL

QIIRVLWKSGNVPHQIMDASGGGGRQTNTFAMNMNASTEGQLRSRRKAAKMLVAVVVMFAFCYFPVHLLSILRKTVGLKNTDGNRAFSLISHWLCYANSAVNPIIYNFMSGKFRKEFHRAFEHCCQRSGGHGFQFSAVYR

KTEKDSGIASRTHSRTDLEIQRVNDFEPRHNRKGTKTSMLLVET

>neuropeptide receptor A16 [Tetranychus urticae] XP_015790996.1

MQLISSYSNISVDIETPIYDNLTSNLICFKEHGNYSSCWFEEELQSNIIPRPYEWIFITLHTLVFIIGLT

GNALICLFVYRNRHLRNVTNYFIVNLAVADFLVILICLPPTVLWDITNTWFFGDLMCKLVVYFQFVSVSV

SVLTLTFISVDRWYAICRPLKFKSTITKTRVAIIFIWISSLAINLPDLIHLQTISPFHRNETIYYTDCVH

DWSEETEKIYQLWIVTTYFILPIILMSVAYHQIAIVLWNKNIPGSCETGQHHHYSNRHSNHNSHNHSQDN

HPDASSIEMNSTIAKRSDCVQGTRSKSKPSYKSSVENSHFGASTNGGNSRTVTKNTSKYPYDSQVTSRQR

VAKMLIAVVIMFGLCYLPVHVLNTLRCFIAIPQNDVISILSLSSHWLCYANSAINPIIYNFMSAKFRKEF

QTTFQNLCGLKFCRKKRRHQGSMESATRRQSNQTQYENINLNSVARYNGS

>metabotropic glutamate receptor [Drosophila melanogaster] NP_001259076.1

MKQKNNNGTILVVVMVLSWSRVVDLKSPSNTHTQDSVSVSLPGDIILGGLFPVHEKGEGAPCGPKVYNRGVQRLEAMLYAIDRVNNDPNILPGITIGVHILDTCSRDTYALNQSLQFVRASLNNLDTSGYECADGSSPQL

RKNASSGPVFGVIGGSYSSVSLQVANLLRLFHIPQVSPASTAKTLSDKTRFDLFARTVPPDTFQSVALVD

ILKNFNWSYVSTIHSEGSYGEYGIEALHKEATERNVCIAVAEKVPSAADDKVFDSIISKLQKKPNARGVV

LFTRAEDARRILQAAKRANLSQPFHWIASDGWGKQQKLLEGLEDIAEGAITVELQSEIIADFDRYMMQLT

PETNQRNPWFAEYWEDTFNCVLTSLSVKPDTSNSANSTDNKIGVKAKTECDDSYRLSEKVGYEQESKTQFVVDAVYAFAYALHNLHNDRCNTQSDQTTETRKHLQSESVWYRKISTDTKSQACPDMANYDGKEFYNNYLLNVSFIDLAGSEVKFDRQGDGLARYDILNYQRQENSSGYQYKVIGKWFNGLQLNSETVVWNKETEQPTSACSLPCEVGMIKKQQGDTCCWICDSCESFEYVYDEFTCKDCGPGLWPYADKLSCYALDIQYMKWNSLFALIPMAIAIFGIALTSIVIVLFAKNHDTPLVRASGRELSYTLLFGILVCYCNTFALIAKPTIGSCVLQRFGIGVGFSIIYSALLTKTNRISRIFHSASKSAQRLKYISPQSQVVITTSLIAIQVLITMIWMVVEPPGTRFYYPD

RREVILKCKIQDMSFLFSQLYNMILITICTIYAIKTRKIPENFNESKFIGFTMYTTCIIWLAFVPIYFGT

GNSYEVQTTTLCISISLSASVALVCLYSPKVYILVFHPDKNVRKLTMNSTVYRRSAAAVAQGAPTSSGYS

RTHAPGTSALTGGAVGTNASSSTLPTQNSPHLDEASAQTNVAHKTNGEFLPEVGERVEPICHIVNK
