# Supplementary material for: Pesticide-induced resurgence in brown planthoppers is mediated by action on a suite of genes that promote juvenile hormone biosynthesis and female fecundity
Source: eLife. 2025 Sep 30;12:RP91774. doi: 10.7554/eLife.91774 (PMC12483516; doi:10.7554/eLife.91774)
Supplement: Supplementary file 4. [file elife-91774-supp4.docx]

**Supplementary File 4 Amino Acid Sequences of Glutamate-Gated Chloride Channels (GluCls) and Acetylcholine Receptors from Diverse Invertebrate Species**

>Glucl transcriptome [Nilaparvata lugens] XP_039283162.1

MAFAINTAIIPLLLYFAQICWCNQPTSSGKINYREKEKQVLDQILGPSSYDARIRPSGVNQTGKHTIVRV

NIFLRSISKIDDYKMEYSVQLTFREQWLDERLKFNDFGGKIKYLTLTEANRVWMPDLFFSNEKEGHFHNI

IMPNVYIRIFPYGSVLYSIRISLTLSCPMNLKLYPLDRQVCSLRMASYGWTTDDLVFLWKEGDPVQVVKN

LHLPRFTLEKFLTDYCNSKTNTGEYSCLKVDLLFKREFSYYLIQIYIPCCMLVIVSWVSFWLDQSAVPAR

VSLGVTTLLTMATQTSGINASLPPVSYTKAIDVWTGVCLTFVFGALLEFALVNYASRSDMHRDNMQKQRRQCELEHAASLEAAADLLQDGGANFPMSDEVLFQKPLVRSGDQTLDKMRQCKVHMQQPQRPNCCRTWLSKFPTRSKRIDVISRITFPLVFALFNLTYWSTYLFRDESDGD

>Glucl transcriptome [Laodelphax striatellus] GenBank: ANN12561.1

MASAIHTAIIPLLLYFAQICWCNQPSSTIKINYREKEKQVLDQILGPSSYDARIRPSGVNQTAGKHTIVR

VNIFLRSISKIDDYKMEYSVQLTFREQWLDERLKFNDFGGKIKYLTLTEANRVWMPDLFFSNEKEGHFHN

IIMPNVYIRIFPYGSVLYSIRISLTLSCPMNLKLYPLDRQVCSLRMASYGWTTDDLVFLWKEGDPVQVVK

NLHLPRFTLEKFLTDYCNSKTNTGEYSCLKVDLLFKREFSYYLIQIYIPCCMLVIVSWVSFWLDQSAVPA

RVSLGVTTLLTMATQTSGINASLPPVSYTKAIDVWTGVCLTFVFGALLEFALVNYASRSDMHRDQMQKQRRQCELEHAASLEAAADLLQDGGTNFPMSDDVLFQKPLVRSGDQTLEKMRQCKVHMQQPHRPNCCRSWLSKFPTRSKRIDVISRITFPLVFALFNLTYWSTYLFRDESDGE

>GABA-gated chloride channel [Sogatella furcifera] GenBank: BAL63029.1

MSRALALVWLAVTITLLRPADRLPPFVHAGTGGGSMLGDVNISAILDSFSVSYDKRVRPNYGGPPVEVGV

TMYVLSISSLSEVKMDFTLDFYFRQFWTDPRLAFRKRPGVETLSVGSEFIKNIWVPDTFFVNEKQSYFHI

ATTSNEFIRIHHSGSITRSIRLTITASCPMNLQYFPMDRQLCHIEIESFGYTMRDIRYKWNEGPNSVGVS

NEVSLPQFKVLGHRQRAMEISLTTGNYSRLACEIQFVRSMGYYLIQIYIPSGLIVIISWVSFWLNRNATP

ARVALGVTTVLTMTTLMSSTNAALPKISYVKSIDVYLGTCFVMVFASLLEYATVGYMAKRIQMRKNRFMAIQKIAEQKQKEAAGHVPGVPGGDPADHAPKQTEVRFKVHDPKAHFKGGTLENTINGRADEEVHLPAPQHLIHPGKDINKLYGITPSDIDKYSRIVFPVCFVCFNLMYWIIYLHISDVVADDLVLLEEDK

>Glucl transcriptome [Tribolium castaneum] NP_001107776.1

MYTHTLIALIVHIIHVTVCTNVKINFREKEKEVLDQILGQGMYDARIRPSGVNGTDGPAIVRVNIFVRSI

SKIDDVTMEYSVQLTFREQWLDERLKFNDFGGRLKYLTLTEASRVWMPDLFFSNEKEGHFHNIIMPNVYI

RIFPYGSVLYSIRISLTLSCPMNLKLYPLDRQICSLRMASYGWTTDDLVFLWKEGDPVQVVKNLHLPRFT

LEKFLTDYCNSKTNTGEYSCLKVDLLFKREFSYYLIQIYIPCCMLVIVSWVSFWLDQGAVPARVSLGVTT

LLTMATQTSGINASLPPVSYTKAIDVWTGVCLTFVFGALLEFALVNYASRSDMHRENMKKQRRQCELEHAASMDATSDLIDTDSNATFAMKPLVRHPGDPMSLEKVRQCEIHMQPARPNCCRSWLSKFPTRSKRIDVISRITFPLVFALFNVIYWSTYLFREEAEES

>Glucl transcriptome [Aethina tumida] XP_049817926.1

MYRHTIFVLILHLMHMSMCTNVKINFREKEKEVLDQILGPGRYDARIRPSGVNGTVVGDGPTIVNINIFL

RSISKIDDYKMEYSVQLTFREQWLDERLKFNDFGGRLKYLTLTEANRVWMPDLFFSNEKEGHFHNIIMPN

VYIRIFPYGSVLYSIRISLTLSCPMNLKLYPLDRQICSLRMASYGWTTDDLVFLWKEGDPVQVVKNLHLP

RFTLEKFLTDYCNSRTNTGEYSCLKVDLLFKREFSYYLIQIYIPCCMLVIVSWVSFWLDQGAVPARVSLG

VTTLLTMATQTSGINASLPPVSYTKAIDVWTGVCLTFVFGALLEFALVNYASRSDMHRENMKKQRRQCELEHAASLDGTSDLIDSDSNATFAMKPLVRHPGDPMNMDKVRQCEIHMQPAPPNCCKSWLSKFPTRSKRIDVISRITFPLVFALFNVIYWSTYLFREETEES

>Glucl transcriptome [Coccinella septempunctata] XP_044765950.1

MITYNFILILLCLLHVTISTNVKINFREKEKEVLDQILGPGRYDARIRPAGANGTGHIGDGPALVHINFF

LRSISKIDDYKMEYSVQLTFREQWQDERLKFNDFGGRLKYLTLTEASRVWMPDLFFSNEKEGHFHNIIMP

NVYIRIHPHGSVLYSIRISLTLSCPMNLKLYPLDRQICSLRMASYGWTTDDLVFLWKEGDPVQVVKNLHL

PRFTLEKFLTDYCNSKTNTGEYSCLKVDLLFKREFSYYLIQIYIPCCMLVIVSWVSFWLDQGAVPARVSL

GVTTLLTMATQTSGINASLPPVSYTKAIDVWTGVCLTFVFGALLEFALVNYASRSDMHRENMKKQRMQCELEHAASLDATSDFIDTESNATFAMVKLKPLVHHPGDPMNLEKVRQCEIHMQPERPNCCRSWLSKFPTRSKRIDVISRITFPLVFALFNVIYWSTYLFREEAEES

>Glucl transcriptome [Plutella xylostella] GenBank: AFO38420.1

MDVLRPSCALFVLFLLYCAHLTECVNAKINFREKEKQILDQILGPGRYDARIRPSGINGTGDAPTLVRVN

LYLRSISKIDDYKMEYSVQLTFREQWLDERLKFNNLGGRLKYLTLTEANRVWMPDLFFSNEKEGHFHNII

MPNVYIRIFPNGNVLYSIRISLTLSCPMNLKLYPLDKQTCSLRMASYGWTTDDLVFLWKEGDPVQVVKNL

HLPRFTLEKFLTDYCNSKTNTGEYSCLKVDLLFKREFSYYLIQIYIPCCMLVIVSWVSFWLDQGAVPARV

SLGVTTLLTMATQSSGINASLPPVSYTKVIDVWTGVCLTFVFGALLEFALVNYASRSDMHRENMKKTRRE

MEAAASMDAASDLLDTDSNATFAMKPLMRGAVLDSKMRQCEVHMAPPRKNCCRLWMSKFPTRSKRIDVISRITFPLVFALFNLAYWSTYLFRDEDEED

>GluCl transcriptome [Helicoverpa armigera] XP_021191008.1

MDIPRPSCALVLVLLFVTHLSECMNGGKINFREKEKQILDQILGPGRYDARIRPSGINGTGYAPTLVHVN

MYLRSISKIDDYKMEYSVQLTFREQWLDERLKFNNLGGRLKYLTLTEANRVWMPDLFFSNEKEGHFHNII

MPNVYIRIFPNGNVLYSIRISLTLSCPMNLKLYPLDKQTCSLRMASYGWTTDDLVFLWKEGDPVQVVKNL

HLPRFTLEKFLTDYCNSKTNTGEYSCLKVDLLFKREFSYYLIQIYIPCCMLVIVSWVSFWLDQGAVPARV

SLGVTTLLTMATQSSGINASLPPVSYTKAIDVWTGVCLTFVFGALLEFALVNYASRSDMHRENMKKARREMEAASMDAASDLLDTDSNTTFAMKPLVRGGVVESKMRQCEIHITPPRKNCCRLWMSKFPTRSKRIDVISRITFPLVFALFNLAYWSTYLFRDEDEEK

>GluCl transcriptome [Bombyx mori] XP_012545491.1

MEFPRRPCALLVLILSYFTQLAICMNSGKINFREKEKQILDQILGPGRYDARIRPSGINGTGDAPTLVRV

NMYLRSISKIDDYKMEYSVQLTFREQWLDERLKFNNLGGRLKYLTLTEANRVWMPDLFFSNEKEGHFHNIIMPNVYIRIFPNGTVLYSIRISLTLSCPMNLKLYPLDKQTCSLRMASYGWTTDDLVFLWKEGDPVQVVKN

LHLPRFTLEKFRTDYCNSKTNTGEYSCLKVDLLFKREFSYYLIQIYIPCCMLVIVSWVSFWLDQGAVPAR

VSLGVTTLLTMATQSSGINASLPPVSYTKAIDVWTGVCLTFVFGALLEFALVNYASRSDMHRENMKKTRREMEAASMDAASDLLDTDSNATFAMKPLVRGGVDTKMRQCEIHISQPRKNCCRLWMSKFPTRSKRIDVISRITFPLVFAIFNLAYWSTYLFRDEEEEK

>GluCl transcriptome [Aedes aegypti] XP_001662897.2

MAPGHYFWAIFYFACLCSASLANNAKVNFREKEKKILDQILGAGKYDARIRPSGINGTDGPAVVRVNIFV

RSISKIDDVTMEYSVQLTFREQWLDERLKFDDIGGRLKYLTLTEANRVWMPDLFFSNEKEGHFHNIIMPN

VYIRIFPYGSVLYSIRISLTLACPMNLKLYPLDRQVCSLRMASYGWTTADLVFLWKEGDPVQVVKNLHLP

RFTLEKFLTDYCNSKTNTGEYSCLKVDLLFKREFSYYLIQIYIPCCMLVIVSWVSFWLDQGAVPARVSLG

VTTLLTMATQTSGINASLPPVSYTKAIDVWTGVCLTFVFGALLEFALVNYASRSADRAADIQRENMKKKR

REMEQVSLDAASDLLDTDSNATFAMKPLVRHPGDPMAMEKLRQCEVHMQAPKRPNCCRTWWSRFPTRQCSRSKRIDVISRITFPLVFALFNLVYWSTYLFREEEED

>GluCl transcriptome [*Musca domestica*] *XP_019892425.1*

MGTGHFFWVIFYFASLCSASLANNAKVNFREKEKKVLDQILGAGKYDARIRPSGINGTENLPTYVYVNMF

LRSISKIDDYKMEYSVQLTFREQWTDERLKFDDIQGRLKYLTLTEANRVWMPDLFFSNEKEGHFHNIIMP

NVYIRIFPNGSVLYSIRISLTLACPMNLKLYPLDRQICSLRMASYGWTTNDLVFLWKEGDPVQVVKNLHL

PRFTLEKFLTDYCNSKTNTGEYSCLKVDLLFKREFSYYLIQIYIPCCMLVIVSWVSFWLDQGAVPARVSL

GVTTLLTMATQTSGINASLPPVSYTKAIDVWTGVCLTFVFGALLEFALVNYASRSDMHKENMKKKRRDLEQASIDAASDLLDTDSNATFAMKPLVRHPGDPLALEKLRQCEVHMQAPKRPNCCKTWLSKFPTRSKRIDVISRITFPLVFALFNLVYWSTYLFREEEDETF

>DrosGluCl [*Drosophila melanogaste*r] GenBank: CAA05260.1

MGSGHYFWAILYFASLCSASLANNAKVNFREKEKKVLDQILGAGKYDARIRPSGINGTDGPAVVRVNIFV

RSISKIDDVTMEYSVQLTFREQWTDERLKFDDIQGRLKYLTLTEANRVWMPDLFFSNEKEGHFHNIIMPN

VYIRIFPNGSVLYSIRISLTLACPMNLKLYPLDRQICSLRMASYGWTTNDLVFLWKEGDPVQVVKNLHLP

RFTLEKFLTDYCNSKTNTGEYSCLKVDLLFRREFSYYLIQIYIPCCMLVIVSWVSFWLDQGAVPARVSLG

VTTLLTMATQTSGINASLPPVSYTKAIDVWTGVCLTFVFGALLEFALVNYASRSGSNKANMHKESMKKKRRDLEQASLDAASDLLDTDSNATFAMKPLVRHPGDPLALEKRLQCEVHMQAPKRPNCCKTWLSKFPTRSKRIDVISRITFPLVFALFNLVYWSTYLFREEEDETF

>GluCl [Formica exsecta] XP_029677035.1

MGACSFLHGGSPGDEPREAGAMWPGVLPLLVLLAVLLHPSRCTQTQTQAKVNFREKEKQVLDNILGPGRYDARIRPSGENGTDGPTIVNVNIFLRSISKIDDYNMEYSVQLTFREQWLDERLRFNDFGGKLKYLTLTEAS

RVWMPDLFFSNEKEGHFHNIIMPNVYIRIFPHGSVLYSIRISLTLSCPMNLKLYPLDRQICSLRMASYGW

TTNDLVFLWKEGDPVQVVKNLHLPRFTLEKFLTDYCNSKTNTGEYSCLKVDLLFKREFSYYLIQIYIPCC

MLVIVSWVSFWLDQSAVPARVSLGVTTLLTMATQTSGINASLPPVSYTKAIDVWTGVCLTFVFGALLEFA

LVNYASRSDMHSDNIKKQFPPSEMEHSSSIDPSSELLEPDGSANFAMKPLVRHPEDSMSMDKLRQCEIHM

QPRKKNCCRSWLSKFPTRSKRIDVISRIFFPIVFAFFNLAYWSTYLFREETDSE

>GluCl [Linepithema humile] XP_012222033.1

MRPGVLPLLVLLAVLLHPSRCSQPAKVNFREKEKQVLDNILGPGRYDARIRPSGENGTDGPAVVRVNIFV

RSISKIDDVTMEYSVQLTFREQWLDERLRFNDFGGRLKYLTLTEANRVWMPDLFFSNEKEGHFHNIIMPN

VYIRIFPNGSVLYSIRISLTLSCPMNLKLYPLDRQVCSLRMASYGWTTNDLVFLWKEGDPVQVVKNLHLP

RFTLEKFLTDYCNSKTNTGEYSCLKVDLLFKREFSYYLIQIYIPCCMLVIVSWVSFWLDQSAVPARVSLG

VTTLLTMATQTSGINASLPPVSYTKAIDVWTGVCLTFVFGALLEFALVNYASRSDMHSDNLKKQFPPSEM

EHSSSIDPTSELLEPDGSANFAMKPLVRHPEDSMSMDKLRQCEIHMQPRKKNCCRSWLSKFPTRSKRIDV

ISRIFFPIVFALFNLAYWSTYLFREEEEGE

>GluCl [Apis cerana] XP_016908712.1

MHALSTCNEYSVQLTFREQWLDERLRFNDFGGRLKYLTLTDASRVWMPDLFFSNEKEGHFHNIIMPNVYI

RIFPNGSVLYSIRISLTLSCPMNLKLYPLDRQVCSLRMASYGWTTDDLVFLWKEGDPVQVVKNLHLPRFT

LEKFFTDYCNSKTNTGEYSCLKVDLLFKREFSYYLIQIYIPCCMLVIVSWVSFWLDQSAVPARVSLGVTT

LLTMATQTSGINASLPPVSYTKAIDIWTGVCLTFVFGALLEFALVNYASRSDMHSDNIEKKYPPSETEQS

TSMDLPSDQVEPDNSSNFAMTGYDGPLQHSLQKPLVRQPEDTMSVDRMQHCELHMQPRKKNCCRSWLSKFPTRSKRIDVISRIFFPIVFAFFNLAYWSTYLFREEEQNE

>GluCl [Apis mellifera] XP_006560233.1

MWPGVLKLLVLLTFLLHPSRCTQGKVNYREKEKEVLDNILGGYDARIRPSGENATDEPTNVMVNIFLRSI

SKINDYNMEYSVQLTFREQWLDERLRFNDFGGRLKYLTLTDASRVWMPDLFFSNEKEGHFHNIIMPNVYI

RIFPNGSVLYSIRISLTLSCPMNLKLYPLDRQVCSLRMASYGWTTDDLVFLWKEGDPVQVVKNLHLPRFT

LEKFFTDYCNSKTNTGEYSCLKVDLLFKREFSYYLIQIYIPCCMLVIVSWVSFWLDQSAVPARVSLGVTT

LLTMATQTSGINASLPPVSYTKAIDIWTGVCLTFVFGALLEFALVNYASRSDMHSDNIEKKYPPSETEQS

TSMDLPSDQVEPDNSSNFAMTGYDGPLQHSLQKPLVRQPEDTMSVDRMQHCELHMQPRKKNCCRSWLSKFPTRSKRIDVISRIFFPIVFAFFNLAYWSTYLFREEEQNE

>GluCl Tetranychus urticae XP_015785428.1

MLFEIAMTLAVFTINLCPSATNRVAINRGSHIDLPNSKSHSNLRTKEKKILDEIIGDGRYDNRIRPSGYN

TSTSDSDSDEGDDGPCVVMVNIFLRSISKISDLDMEYSVQITFREEWRDERLQYNDNNEQIKFLTLTDPN

RIWKPDLFFSNEKEGHFHTIIMPNVLLRIYPDGSVLYSIRISLLLACPMDLKYYPLDEQECFMRMASYGY

TTDDLEFRWKDGDPVQITSNLHLPRFALQKYKTAYCTSRTNTGEYSCLKVYLDFKREFSYYLIQIYMPCC

MLVIVSWVSFWLDPNAIPARVSLGVTTLLTMATQISGINASLPPVSYIKAIDVWTGVCLAFVFGALLEFA

LVNYASRSDAHRHAREFNGLRYQRRWDRDGNVISDETSYALRPLVIKGSEYSNKNIFSRWWSKFPTRSKR

IDVVSRIFFPLMFCLFNLVYWVTYLFRHKRDKNVY

>GluCl  Parasteatoda tepidariorum XP_015913139.2

MAKYLILGLVLSMFLLSSAEVNDRRLEVERNILSTIFEKYDRRIRPAGLNNTGPVVVVVDTYVRSINNID

DVNMVYDTQLTFRQTWHDDRLKYNNRNFNYVTLTDPEMLWTPDLFFSNEKSGHFHNMLKPNVLIRIHPDGKILYSLRISLRLSCPMSLDRYPLDNQVCSMVAASYGYTTDDIEFVWKSINPVQVSKNLFLPQFQLKSYTQDYCTSTTNTGTYSCIKIDLVLSRNSFYYMIQFYMPTACMVLLSWLVFWLNPKYTTIRFGIILGVLISSII

ITTSIHSRTPQVSYTKASDVWIGVCVCFIFSVILELCLVVNFLSKEEYSRLQNQLTADELLGDNSIRIDD

VGKASQVRNIQSAPLKWLNKYSSTAQRIDVFSRFLFPALFAFFNLVYWLKYAA

>GluCl Caenorhabditis elegans NP_507090.1

MATWIVGKLIIASLILGIQAQQARTKSQDIFEDDNDNGTTTLESLARLTSPIHIPIEQPQTSDSKILAHL

FTSGYDFRVRPPTDNGGPVVVSVNMLLRTISKIDVVNMEYSAQLTLRESWIDKRLSYGVKGDGQPDFVIL

TVGHQIWMPDTFFPNEKQAYKHTIDKPNVLIRIHNDGTVLYSVRISLVLSCPMYLQYYPMDVQQCSIDLA

SYAYTTKDIEYLWKEHSPLQLKVGLSSSLPSFQLTNTSTTYCTSVTNTGIYSCLRTTIQLKREFSFYLLQ

LYIPSCMLVIVSWVSFWFDRTAIPARVTLGVTTLLTMTAQSAGINSQLPPVSYIKAIDVWIGACMTFIFC

ALLEFALVNHIANKQGVERKARTEREKAEIPLLQNLHNDVPTKVFNQEEKVRTVPLNRRQMNSFLNLLET

KTEWNDISKRVDLISRALFPVLFFVFNILYWSRFGQQNVLF

>GluCl Caenorhabditis elegans NP_495489.2

MKAQLYVSVLLALLVSSTAKKSKTKSCKRTAFSRHTTNYQAWREQMTVCDLLQDYDAAVRPSGRTPYNDTRGAVMVTTSLNIRSISAVSEKNMEFVAQFRFRQEWYDDRLRFIEHQGLLSSDYRNFEFIHVARDQSLWIPDTFFQNEKNGWYHMLNQENRFLKIRSDGKLIYDRRLTLHLACSMHLSRYPMDHQNCEIAFASYAYTTADIEYIWDVPAIQIHEGANGALPNFEIASFKNASCTSKTNTGTYSCLKVEIRLNRVFSFFLLQLYIPSSMLVG

VAWVSYWIDWKSTAARVPLAIVTLLTMITTSHAINSNLPPVSYAKSIDIWVGACVVFIFFSLIEYAVVNY

VGILDEHRQMKKAACNRSRLSNVIENDNFGESLQSLTFSPQEKKRLIRRRPKKNMEMQEGDFEAIEMVDR

GPPRSAGLMEEGWTFHDTTDLVYIGQRKRVELVRWCSVLSSRGRAERIDIIARIIFPLAFILFNFAYWSI

YLEEEDPDES

>GluCl Trichinella spiralis XP_003375388.1

MHFPTMHYSLQHFVTVIILSVVYDRPLNVESLDFNNWAKAQMSWKNEYSSSVWKFRPWREQYIIRELLKTYDIKTRPQVSKSPTTDSTSGPDRGEPVVVHTALMLQTINSVDEINMASEFKARFRFEQRWTDERLNFHRF

NDENGKKIENVHLAYDQVIWKPDTFFQNEKSGTFHVVDQPNCFVKIFPDGKVLYNVRIGMTFSCVMNLHNYPMDTQECVIDFASYGYTTEDIIYVWEETPIHVDPQVTSVLPNMAIKAITNGTCTSKTNTGEYSCLRISL

IFERQFSFFLLQLYIPSSLLVVVSCLSYWIDWRASAGRMLLTIVTLLTLITQHYSVSANLPPVSYATAID

VWVGACVVFVFTSLVEYAFVNYIGLIQQQKRIASSYPFQRLSNVQQDVFENLCQQSTSGEHHSSPSFHDE

KYDKYSYFHYFFHGLRSRKNSRRQLSTSRTQAVDEFNNTESLIINNPEFSAEEPCSCVMSQRAQRRLYHQ

LHLPALDTDFYFLWNDRAEKIDSIFRYLIPICFFFFNIVYWSVYYIPVMQAKMKNQFSAR

>acetylcholine receptor Nilaparvata lugens XP_039291748.1

MASGETEAGCQQFLSNFCLVWLAASVIMLRTLLQCIQHLRTSLLRKLKTVHSKILCSLNVCSNFIHTLDV

RSGTNNVRSDLLATLDVRSSFSSTKHVRSDLLATLDVCSGTKHVRSNLLATLDMRSRFLSTKHVRSNLLS

TLDVRSSLSSTLRNVPSEISQSVDVSRTSHVRSSHPRTLAAALMGVCVVLSCMPFAADANPEAKRLYDDL

LSNYNRLIRPVGNNSDRLTVKMGLRLSQLIDVNLKNQIMTTNVWVEQEWNDYKLKWNPDEYGGVDTLHVPSEHIWLPDIVLYNNADGNYEVTIMTKAILHHTGKVVWKPPAIYKSFCEIDVEYFPFDEQTCFMKFGSWTYDGYHVDLRHMSQSPDSDTIDVGIDLQDYYLSVEWDIMRVPAVRNEKFYSCCEEPYPDIIFNITLRRKTLFYTVNLIIPCVGISFLSVLVFYLPSDSGEKVSLCISILLSLTVFFLLLAEIIPPTSLTVPLLGKYLLFTMV

LVTLSVVVTIAVLNVNFRSPVTHKMRPWVHRLFIQVLPKVLLIDRPKKEDSIDEDGAEDDLDKNVDDGNN

IFTGVFDVPAEIDKYLGGYGSSKRFSADYDIPSLPPSRYDSGGPTTTMVPCFGGVGGADPPLPLPLPSAD

DDLFSPGVGVGGVESPTFEHHHDQPITMEKTIKDAKFIAQHVRNKDKFENVIEDWKYVAMVLDRLFLWVFTLACVLGTALIILQAPSLYDTTKPIDILYSKIAKKKMLAMMGPEED
